# Supplementary material for: Personality affects dynamics of an experimental pathogen in little brown bats
Source: R Soc Open Sci. 2020 Sep 16;7(9):200770. doi: 10.1098/rsos.200770 (PMC7540777; doi:10.1098/rsos.200770)
Supplement: Supplementary figures and tables [file rsos200770supp1.docx]

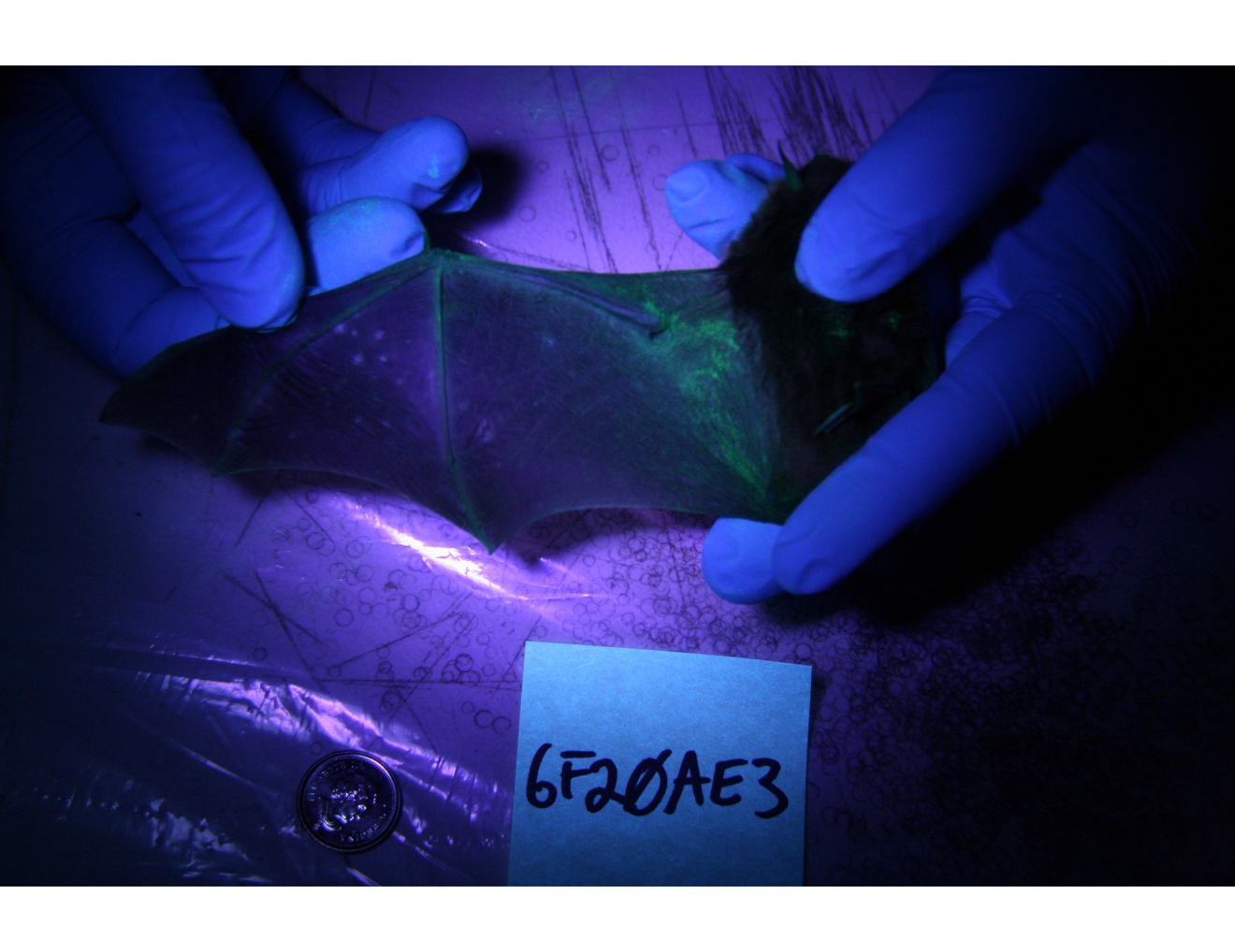


**Figure S1.** Photograph of UV-powder on left dorsal wing surface of a little brown bat (Myotis lucifugus). Note green powder adjacent to body, along the humerus and 5^th^ metacarpal.


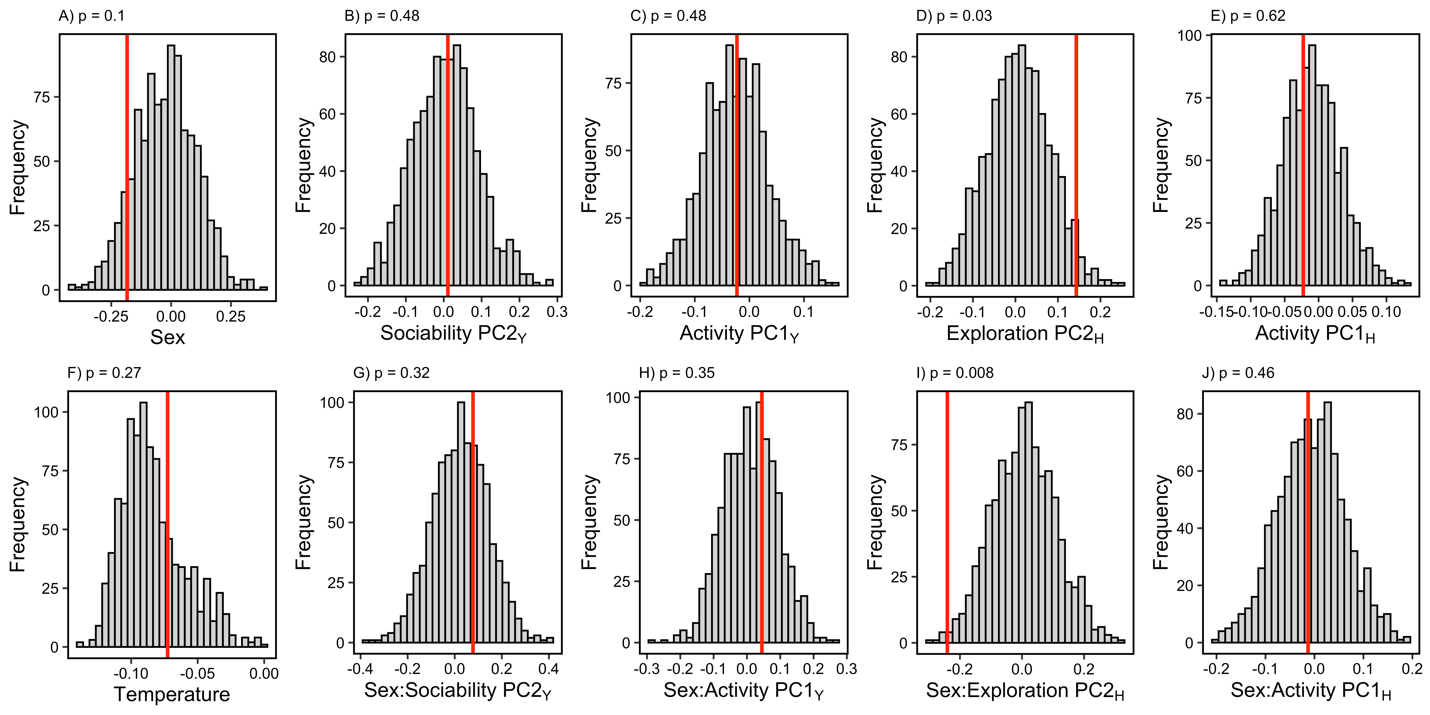


**figure S2:** distribution of permuted coefficients in a linear mixed effects model. Within each iteration of the permutation, we swapped observed values of infection intensity among individuals in the trial and re-tested the relationship between covariates (i.e. sex, temperature at dawn, activity, exploration and sociability). Red lines represent the observed coefficient and one-sided p-values are presented for each test as well as in Table 3 of the main text.


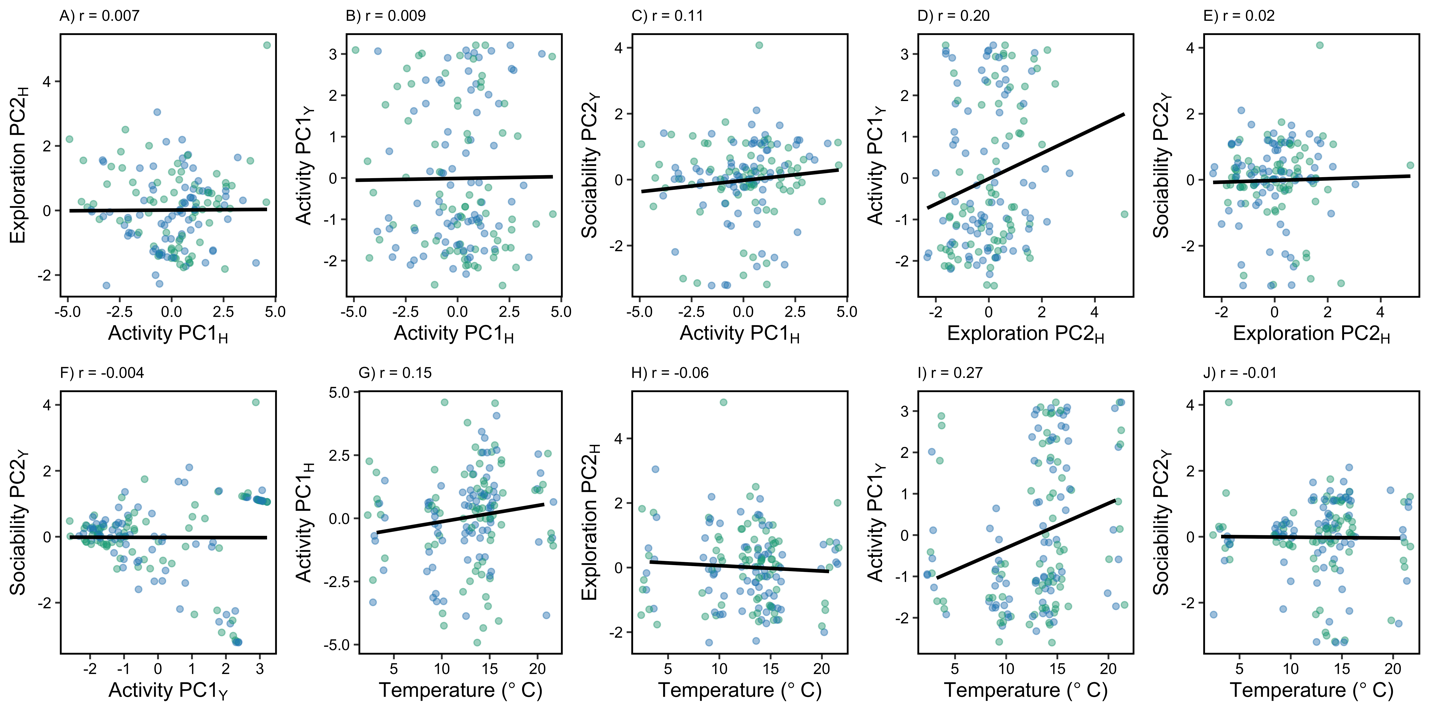


**Figure S3:** pairwise relationships between covariates included in the global linear mixed model where blue points represent male bats and green points represent female bats. All Pearson correlation coefficients were non-significant except the relationship between Activity PC1_Y_ and temperature. Despite this correlation variance inflation factors were <2.2, so all covariates were included in the global model.

**Table S1.** Summary statistics for each of ten trials where a single randomly selected bat was ‘infected’ with UV-fluorescent powder and released into a flight tent with 14 or 15 naïve bats. Standard deviation is included with average intensities of ‘infection’ for females and males.

| **Trial** | **Sex of originally infected bat** | **Number of naïve bats** | **Average intensity of infected females** | **Average intensity of infected males** |
| --- | --- | --- | --- | --- |
| 1 | F | 15 (F = 7; M = 8) | 0.153 ± 0.044 | 0.095 ± 0.081 |
| 2 | F | 14 (F = 7; M = 7) | 0.072 ± 0.053 | 0.039 ± 0.024 |
| 3 | M | 15 (F = 8; M = 7) | 0.044 ± 0.048 | 0.077 ± 0.047 |
| 4 | F | 15 (F = 7; M = 8) | 0.118 ± 0.046 | 0.105 ± 0.069 |
| 5 | M | 15 (F = 8; M = 7) | 0.117 ± 0.072 | 0.095 ± 0.155 |
| 6 | F | 15 (F = 6; M = 9) | 0.027 ± 0.013 | 0.049 ± 0.027 |
| 7 | M | 15 (F = 8; F = 7) | 0.186 ± 0.107 | 0.219 ± 0.125 |
| 8 | F | 15 (F = 6; M = 9) | 0.177 ± 0.070 | 0.149 ± 0.054 |
| 9 | M | 15 (F = 9; M = 6) | 0.459 ± 0.151 | 0.354 ± 0.133 |
| 10 | F | 14 (F = 6; M = 8) | 0.326 ± 0.08 | 0.275 ± 0.106 |

**Table S2.** Summary of results for principal component analysis (PCA) of behavioural responses of little brown bats for a modified hole-board test (n = 160). Bold numbers reflect strong variable loadings on each component (PC loading > |0.40|). See ‘Quantifying personality’ section of the main text methods for description and note that PC3 is presented here, but not included in subsequent analyses. Bartlett’s test indicated there were significant correlations among variables in this dataset (χ^2^ = 1146 p < 0.001), while the KMO test suggested sampling adequacy was appropriate (MSA = 0.54).

| **Behavioural Variables** | **PC1**  **(Activity)** | **PC2**  **(Exploration)** | **PC3** |
| --- | --- | --- | --- |
| Line Crossing | **0.50** | –0.03 | 0.05 |
| Locomotion | **0.48** | –0.23 | –0.11 |
| Flight Attempts | **0.42** | –0.04 | 0.11 |
| Echolocation | –0.47 | 0.26 | –0.12 |
| Number of Head Dips (all holes) | 0.27 | **0.55** | 0.13 |
| Latency to Head Dip (all holes) | –0.21 | **–0.54** | 0.04 |
| Latency to Enter | 0.03 | **0.51** | 0.20 |
| Grooming | 0.10 | 0.15 | **0.95** |
| Standard Deviation | 1.85 | 1.17 | 0.99 |
| % Total Variation | 42.6 | 17.2 | 12.5 |

**Table S3.** Summary of results for principal component analysis (PCA) of behavioural responses of little brown bats in the first trial for a modified Y-maze test (n = 160). Bold numbers reflect strong variable loadings on each component (PC loading > |0.40|). See ‘Quantifying personality’ section of the main text methods for description. Bartlett’s test indicated there were significant correlations among variables in this dataset (χ^2^ = 930, p < 0.001), while the KMO test suggested sampling adequacy was appropriate (MSA = 0.62).

| **Behavioural Variables** | **PC1**  **(Activity)** | **PC2**  **(Sociability)** |
| --- | --- | --- |
| Line Crossing | **0.52** | –0.08 |
| Locomotion | **0.54** | –0.12 |
| Echolocation | **–0.53** | 0.16 |
| Grooming | –0.07 | –0.23 |
| Relative time spent within 10 cm of stimulus bat | –0.39 | **–0.53** |
| Latency to enter 10 cm of stimulus bat | –0.04 | **0.79** |
| Standard Deviation | 1.77 | 1.19 |
| % Total Variation | 52.0 | 23.7 |
